# B7-H4 overexpression contributes to poor prognosis and drug-resistance in triple-negative breast cancer

**DOI:** 10.1186/s12935-018-0597-9

**Published:** 2018-07-13

**Authors:** Ling Wang, Chao Yang, Xin-bo Liu, Li Wang, Fu-biao Kang

**Affiliations:** 1 Department of Orthopedic Oncology, the Third Hospital of Hebei Medical University, Shijiazhuang, Hebei People’s Republic of China; 2 Department of General Surgery, the Fourth Hospital of Hebei Medical University, Shijiazhuang, Hebei People’s Republic of China; 3 Department of Thoracic Surgery, the Fourth Hospital of Hebei Medical University, Shijiazhuang, Hebei People’s Republic of China; 4 Department of Pathology, the Fourth Hospital of Shijiazhuang, Shijiazhuang, China; 5 Department of Liver Diseases, Bethune International Peace Hospital, Shijiazhuang, Hebei People’s Republic of China

**Keywords:** Triple-negative breast cancer, B7-H4, Proliferation, Apoptosis, Chemoresistance

## Abstract

**Background:**

The expression of the immunoregulatory protein B7-H4 has been reported in many types of cancer, including breast cancer. However, its role in triple-negative breast cancer (TNBC), especially its correlation with patients’ prognosis and chemoresistance remains unclear.

**Methods:**

The expression of B7-H4 in TNBC tissues and cell lines were measured with Real-Time PCR and western blotting. 65 cases of TNBC tissue samples and adjacent non-tumor tissue samples were analyzed by immunochemistry to demonstrate the correlation between the B7-H4 expression and clinicopathological characteristics. In vitro studies assessed mAb MIH43 alone and in combination with transfecting B7-H4 siRNA on the growth of chemosensitive and chemoresistant TNBC cell lines by CCK-8 and apoptotic enzyme-linked immunosorbent assay (ELISA).

**Results:**

B7-H4 expression was detected positive in 59 of 65 (90.8%) different stage TNBC patients, especially in the samples of recurrence TNBC patients after receiving neoadjuvant chemotherapy treatment. Survival curves showed that patients with B7-H4 overexpression had significantly shorter survival and recurrence time than those with low B7-H4 expression (*p *< 0.005). Univariate and multivariate COX regression analysis demonstrated that B7-H4 was an independent predictor for advanced tumor stage. The monoclonal antibody of B7-H4 has the potential anti-proliferative effects on inhibiting the chemoresistant TNBC cell lines and increasing the sensitivity of TNBC cell lines to doxorubicin, paclitaxel or carboplatin. RNAi-mediated silencing of B7-H4 in TNBC cells enhanced drug-induced apoptosis via inhibiting PTEN/PI3K/AKT pathway, whereas reexpression of B7-H4 in B7-H4 knockdown and low B7-H4 expressing cells increased the phosphorylation of PI3K and AKT along with restoration of PETN expression.

**Conclusions:**

Our data show that B7-H4 is a biomarker indicative of a poor prognosis in TNBC patients and at least partially downregulated in chemoresistance via PTEN/PI3K/AKT pathway. Targeting B7-H4 might provide an attractive therapeutic approach specifically for TNBC patients.

**Electronic supplementary material:**

The online version of this article (10.1186/s12935-018-0597-9) contains supplementary material, which is available to authorized users.

## Background

Breast cancer is a heterogeneous disease and TNBC is one of the most biologically aggressive subtypes [1, 2]. TNBC represents 10–20% of breast cancer and the mainstay of therapy remains chemotherapy and no targeted therapy is currently available for it. Although TNBC patients are sensitive to adjuvant chemotherapy, the prognosis is very poor and prone to cause chemotherapy-resistant and recurrence within 3-years [3, 4]. Therefore, to identify potential therapeutic targets, a better understanding of the biology of TNBC is needed. There is increasing evidence showed that adding targeted therapies to adjuvant chemotherapy may increase the sensitivity of residual disease, which will reduce the dose of chemotherapy necessary to kill the remaining tumor cells, thereby minimizing the toxicity of the prolonged treatment [5, 6].

Upregulation of immune inhibitory molecules such as co-regulatory ligands/receptors and tolerogenic enzymes by cancer cells allow tumors escape from immune attack [7, 8]. Immune checkpoint inhibitors such as PD-1 and CTLA-4 have shown prominent and durable responses in diverse malignancies [9, 10]. B7-H4 is one of the most recently identified members of the B7 homologue family of immune co-regulatory molecules and shown to exert an immunosuppressive effect in regulation of T cell immunity through the inhibition of T-cell function, such as activation, proliferation, cytokine production and cytotoxic activity [11–13]. Recently, many studies have reported that B7-H4 is implicated in various types of human tumors, including renal cell carcinoma, ovarian cancer, gastric cancer, and breast cancer, where it plays an important part in tumor progression and is associated with a poor prognosis [14–17]. However, whether and how the engagement of B7-H4 by counter molecules affects the fate of B7-H4-expressing cells is poorly understood.

In this study, we investigate the prevalence and prognostic value of B7-H4 on TNBC with the clinicopathological characteristics and patients’ outcome. In addition, we also demonstrate the potential role of B7-H4 played in TNBC cells after received chemotherapy treatment. The aim of our study was to further clarify whether B7-H4 could be identified as a reliable marker for the appropriate selection of high-risk patients eligible for personal-designed targeted therapeutic agents. And whether blocking B7-H4 could be an alternative approach to avoid residual cancer cells become drug-resistant.

## Materials and methods

### Microarray data sources

Microarray datasets of invasive breast carcinoma (IDC) were downloaded from the Cancer Genome Atlas: Invasive Breast Carcinoma Gene Expression Data, 2011, (http://tcga-data.nci.nih.gov/tcga/). These data were accessed via the ONCOMINE Cancer Profiling Database and used to investigate B7-H4 expression in IDC and non-IDC of breast cancer.

### Patients and diagnostic criteria

Tissue samples (frozen and FFPE) were separately obtained from 65 untreated Chinese women diagnosed with TNBC at the Fourth Hospital of Hebei Medical University from January 2005 to December 2010. All cases were reviewed by two experienced pathologists, and assessed in accordance with the criterion of the world health organization (WHO) breast cancer pathology [18]. In our study, TNBC diagnosis was determined and based on negative test results in ER, PR, and HER-2 from biopsy samples. The baseline clinicopathological data including age, tumor size, lymph node metastasis, distant metastasis, tumor grade, TNM stage, histological type, and survival information were retrieved. The TNM stage was determined based on the American Joint Committee on Cancer (AJCC) criteria and the histological grade was assessed according to the modified Bloom-Richardson classification. For these recurrence patients, the initial symptoms for patients are different, including neurological symptoms, abdominal symptoms, lung symptoms and bone symptoms, etc. Some patients discover the recurrence by themselves, while others were detected by physician examination. Breast cancer recurrence was diagnosed by radiography or discovered by tumor CA 15-3 elevation or/and CAE elevation in patients. This study was reviewed and approved by the Review Board of Fourth Hospital of Hebei Medical University. All patients gave their written and informed consent.

### Cell lines and cell culture

TNBC cell lines MDA-MB-231, MDA-MB-435, MDA-MB-468 and non-TNBC cells MCF-7 and MCF-10F were obtained from the Cell Bank of the Chinese Academy of Sciences (Shanghai, China) and cultured according to the instructions from American Type Culture Collection (ATCC). Cell lines were all maintained in suitable medium supplemented with 10% fetal bovine serum and 1% penicillin/streptomycin. For MDA-MB-435/DOX (doxorubicin) cell culture, DOX was added in the RPMI 1640 medium to achieve the final DOX concentration of 2 μg/mL [19]. All cell lines were maintained in antibiotic-free medium at 37 °C in a 5% CO_2_ atmosphere and routinely screened for mycoplasma contamination.

### Immunohistochemical (IHC) staining

For all IHC analyses, FFPE tumor samples were prepared for 5 μm slices and antigen retrieval was performed by citrate buffer solution (pH = 6) for 5 min. Endogenous peroxidase was blocked with 0.3% H_2_O_2_ in methanol for 15 min, and all slides were heated to 100 °C for 20 min and then cooled at room temperature. Non-specific binding sites were blocked by 10% bovine serum albumin (BSA) for 30 min. The slides were then washed in phosphate-buffered saline, and primary antibody against B7-H4 (diluted in 1:500) was applied for overnight incubation. On the next day, anti-mouse/rabbit IgG were added for 1 h incubation at 37 °C. Color development with DAB substrate was performed and counterstaining with hematoxylin was conducted.

### Evaluation of immunohistochemical staining

Evaluation of B7-H4 staining in tumor cells was evaluated by authorized pathologists who had no knowledge of the patients’ clinical status and outcome. B7-H4 expression scores were given separately for the stained area and for the intensity of staining. Quantification was made as follows; ≤ 25% of the cancer cells: 1, > 26% to ≤ 50% of the cancer cells: 2, > 51% to ≤7 5% cancer ce cancer cells: 3, > 76% of the cancer cells: 4; intensity of staining: absent/weak: 1, moderate: 2, strong: 3. Each section had a final grade that derived from the multiplication of the area and intensity scores. The final B7-H4 staining score was calculated using the percent of positive cell score × staining intensity score ranging 0–12. The final scores ≤ 4 was classified as tumors with low B7-H4 expression, whereas sections with a final score of > 4 were classified as tumors with high B7-H4 expression.

### In vitro growth inhibition

Cells (1 × 10^4^ cells) were initially plated in triplicate in 96-well culture plates. Twenty-four hours later, the medium was replaced with fresh medium with or without different drugs and incubated for indicated time. Cell viability was determined by CCK-8. In order to detect the effects of B7-H4 in cell proliferation, B7-H4 mAb (MIH43, ab110221) and its isotype control mAb IgG1 (RM106, ab190481) were purchased from abcam company and perform the cell growth assay. The absorbance value at 450 nm was read using a microplatereader (Bio-Rad, CA, USA).

### Transient transfection of B7-H4 overexpressing or silencing plasmid

To further analyze the role of B7-H4 in TNBC, we transfected TNBC cells with the B7-H4 cDNA (Origene, Inc) or B7-H4 siRNA (Origene, Inc) using Lipofectamine^3000^ (Invitrogen, CA). In brief, about 3 × 10^5^ cells were seeded per well in a 6 well plate. After 24 h, the cells were transfected with 1.5 μg of cDNA or siRNA plasmid for 6 h, and the media were replaced with fresh growth medium. At 48 h after transfection, cells were harvested for analysis. The silencing or overexpressing effects of B7-H4 in TNBC cells were detected and confirmed (Additional files 1, 2).

### RNA extraction, reverse transcription and real-time RT-PCR

Total RNA was extracted from cell lines and freshly frozen samples with TRIzol reagent (Invitrogen, USA) and was reverse-transcribed with the first strand cDNA synthesis kit (Invitrogen). Real-time PCR reactions were conducted using SYBR Premix Ex Taq II (Takara). Reverse transcriptase was used as the negative control, and glyceraldehyde-3-phosphate dehydrogenase (GAPDH) was used as the endogenous control. All experiments were repeated three times. The PCR primers used in this study were as follows: B7-H4 (F5′: AGGGAGTGGAGGAGGATACAG, R5′: GCAGCAGCCAAAGAGACAG), GAPDH (F5′: CACCATCTTCCAGGAGCGAG, R3′: GACTCCACGACGTACTCAGC).

### Quantification of apoptosis by ELISA kit

An apoptosis ELISA kit (Roche Diagnostics Co.) was used to quantitatively measure cytoplasmic histone–associated DNA fragments. After treatment with different concentration of DOX or B7-H4 mAb MIH43 (5 or 10 μg/mL) up for 72 h, cells were analyzed by following manufacturer’s protocol. Each experiment was repeated three times.

### Western blot analysis

Protein from TNBC tissue samples and cells were separated by SDS-PAGE and then electro-transferred onto nitrocellulose membrane (Bio-Rad). The primary antibodies used included antibodies to B7-H4, PARP, Caspase-3, Caspase-7, Caspase-9, PETN, p-PI3K, PI3K, p-AKT, AKT and GAPDH were purchased from Abcam company. Membranes were probed with indicated antibodies by following the manufacturer’s protocol, and immunoreactive bands were visualized by using ECL Western Blotting Substrate (Pierce Biotechnology, Inc.). Each experiment was repeated three times.

### Statistical analysis

Results are reported as mean ± SD. All the experimental data were analyzed by the SPSS 20.0 statistical software package. The Mann–Whitney U test, χ^2^ test, Pearson Chi square test or Spearman rho test were performed for comparative statistical evaluations among groups and for correlation analysis with histological and clinical parameters (age, gender, tumor stage, tumor grade, and postoperative survival). Survival periods were counted in months from the date of first visit to date of death or last follow-up before study closure. We used Kaplan–Meier method to estimate the overall survival for low and high levels of B7-H3 expression. A *p* value < 0.05 was considered as statistically significant.

## Results

### Elevated B7-H4 expression associated with human TNBC progression

**Fig. 1:**
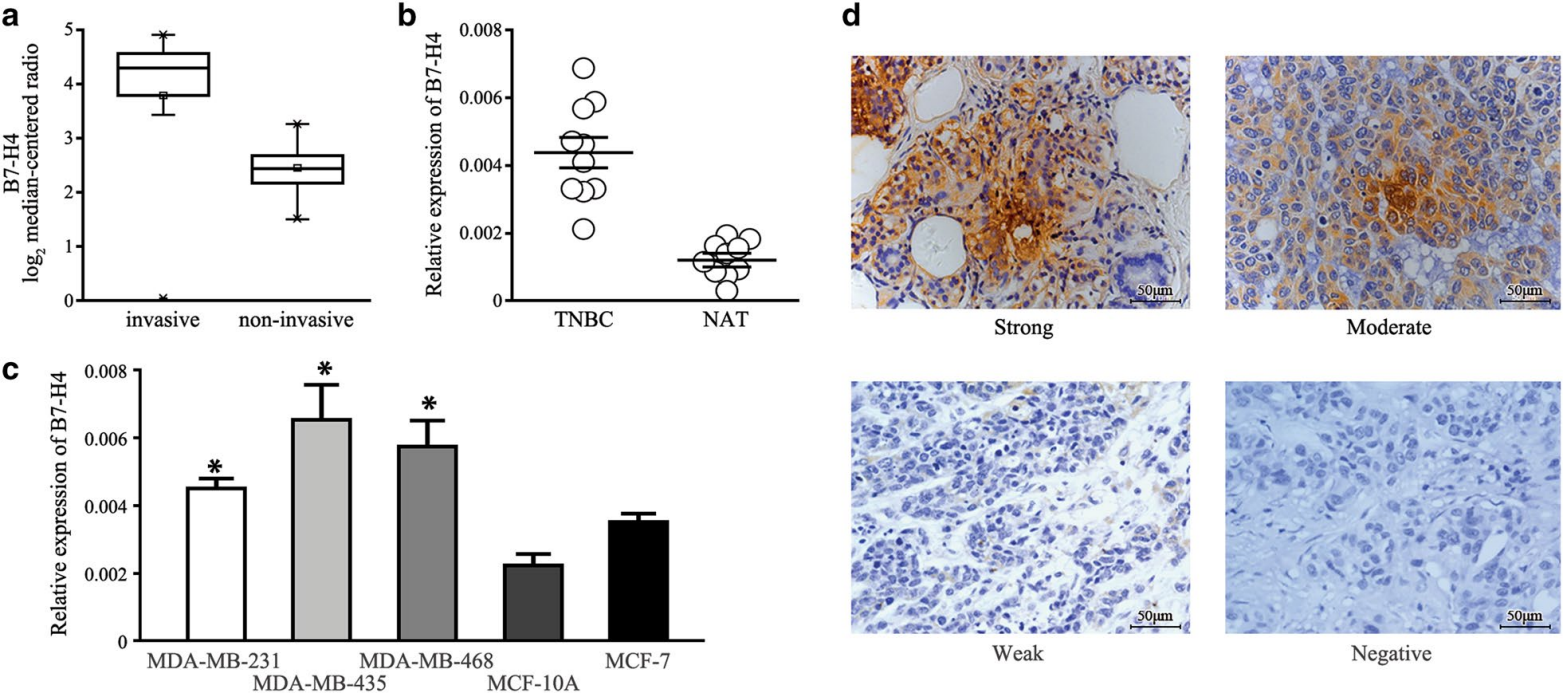
B7-H4 is highly expressed in invasive breast cancer and particularly overexpressed at high levels in TNBC subtype. **a** B7-H4 is significantly overexpressed in invasive breast cancer tissue compared with non-invasive breast cancer tissue samples from the public breast cancer microarray database. **b** The expression level of B7-H4 mRNA in TNBC samples compared with adjacent non-tumor tissue samples. **c** The expression level of B7-H4 mRNA in various breast cancer cell lines was evaluated using real-time PCR. The data are presented as the mean ± SD. **d** B7-H4 immunostaining in TNBC tissues and normal breast tissue. ×200 magnification

To evaluate the expression of B7-H4 in human TNBC, we used the publicly available cancer microarray database to estimate the status of B7-H4 transcript. The result demonstrated differential B7-H4 expression was significantly increased in invasive breast carcinoma compared with non-invasive breast carcinoma tissues (Fig. 1a). To further testify the analytical result, 10 fresh TNBC tissue and adjacent non-tumor adjacent tissue (NAT) together with TNBC cell lines were also collected. Following RNA extraction, qRT-PCR confirmed the high presence of B7-H4 mRNA in all these TNBC samples with transcript levels, and MDA-MB-435 cells showed baseline high B7-H4 expression among all the tested cells (Fig. 1b, c).

In addition to the transcript level of B7-H4 expression, we next evaluated B7-H4 expression at the protein level via immunohistochemical staining. Figure 1d results showed that B7-H4 expression was detected positive in 59 of 65 (90.8%) patients with different stage TNBC who had not received neoadjuvant chemotherapy. In all positive cases, B7-H4 expression was present diffusely throughout the cytoplasm with a pronounced membranous component. According to the staining intensity, there were 6 (9.2%) cases with no tumor B7-H4 intensity, 7 (10.8%) cases with weak tumor B7-H4 intensity, 25 (38.5%) with moderate intensity, and with 27 (41.5%) marked intensity. According to their positive immunoreactivity staining area, a total of 83.1% of tumor samples were high B7-H4 expression, while 16.9% showed a lower degree of B7-H4 staining. However, there was limited expression of B7-H4 in normal breast tissues.

Pertinent clinicopathological findings of the enrolled patients are summarized in Table 1. High expression level of B7-H4 in TNBC was more common in IDC compared with non-IDC subtype and was associated with advanced TNM stage (*p *= 0.006) and likely to develop metastasis (*p *= 0.001) and recurrence (*p *= 0.003). However, B7-H4 expression was not associated with age (*p *= 0.612), menopausal status (*p *= 0.425), tumor size (*p *= 0.055) histological subgroups (*p *= 0.051). Surprisingly, we noticed that there was a reverse correlation between the expression of B7-H4 and androgen receptor (AR, r = − 0.317, *p *= 0.004). Taken together, these data confirmed that B7-H4 expression might be functionally important in tumor progression and metastasis in TNBC.

**Table 1:** Correlation between B7-H4 expression and clinicopathological parameters in TNBC

Parameters	n	B7-H4 expression		*p*	B7-H4 intensity			*p*
		Low	High		Weak	Moderate	Strong	
Age								
≤ 50	30	5	25	0.612	4	14	12	0.664
> 50	29	5	24		3	11	15	
Menopausal								
Premenopausal	30	6	24	0.425	5	13	12	0.440
Postmenopausal	29	5	24		2	12	15	
Tumor histology								
IDC	45	10	35	0.051	7	21	17	0.059
Non-IDC	14	1	13		0	4	10	
Tumor size								
≤ 2	4	3	1	0.055	1	2	1	0.113
2–5	42	7	35		6	20	16	
> 5	13	0	13		0	3	10	
Tumor grade								
I–II	26	9	17	*0.002*	7	12	7	*0.004*
III	33	1	32		0	13	20	
TNM stage								
I	3	3	0	*0.006*	1	2	0	*0.003*
II	41	7	34		6	22	13	
III	15	0	15		0	1	14	
Metastasis								
Yes	21	9	12	*0.001*	7	10	4	*0.001*
No	38	2	36		0	15	23	
Ki-67								
+	8	4	4	0.053	3	3	2	0.062
++	29	6	23		4	13	12	
+++	22	2	20		2	7	13	
AR								
−	38	3	35	*0.007*	2	11	25	*0.003*
+	21	9	12		6	10	5	
Recurrence								
Yes	25	9	16	*0.003*	7	10	8	*0.002*
No	34	4	30		2	13	19	

Numbers in italic indicate statistical significance

### Elevated B7-H4 expression correlated with poor survival of human TNBC patients

**Fig. 2:**
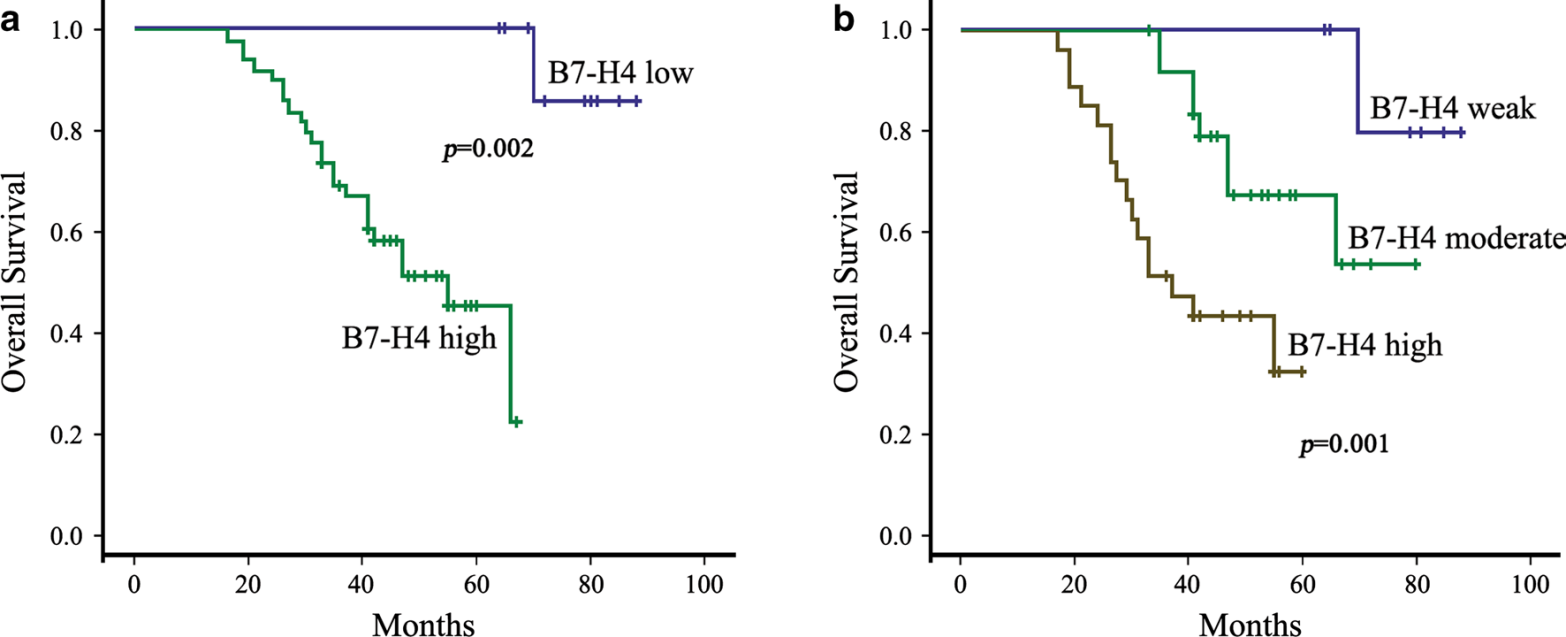
Overall survival of 65 TNBC patients stratified by B7-H4 protein expression levels. **a** Kaplan–Meier plots of overall survival in TNBC patients from the whole data sets stratified by B7-H4 expression levels. **b** Kaplan–Meier plots of overall survival in TNBC patients from the whole data sets stratified by B7-H4 expression intensity

Cumulative survival time analyzed by Kaplan–Meier method showed that patients with high B7-H4 expression had significantly shorter survival times (*p *= 0.002, Fig. 2a), 24 (48.9%) of TNBC patients with high B7-H4 expression died at the time of study, compared with 1 (9.1%) TNBC patients with low B7-H4 expression after diagnosis (*p *= 0.003). Moreover, the average time to recurrence was 48.1 months for patients with low B7-H4 expression, compared to 27.7 months for patients with high B7-H4 expression (*p *= 0.014).

In univariate analysis, larger tumor size, lower tumor grade, advanced TNM stage, high Ki-67, AR, B7-H4 expression, B7-H4 intensity and lymph node positivity were associated with shorter overall OS, while other clinical pathological characteristics, such as age, histological subtype did not influence the prognosis. In the multivariate analysis, B7-H4 staining intensity is an independent prognostic factor for OS in TNBC patients (RR: 0.313, 95% CI 0.114–0.857, *p *= 0.024). Results from the univariate analysis and final multivariate Cox regression model are presented in Table 2. Therefore, these data suggested that weaker B7-H4 intensity is correlated with better prognosis or survival in TNBC.

**Table 2:** Univariable and multivariable analysis for of TNBC overall survival

Characteristics	Univariate		Multivariate	
	HR (95% CI)	*p* value	HR (95% CI)	*p* value
Tumor size	2.418 (1.118–5.228)	0.025	0.393 (0.118–1.311)	0.314
Tumor grade	7.464 (2.493–22.350)	0.011	0.316 (0.041–2.446)	0.270
TNM stage	4.433 (1.947–10.093)	0.032	0.290 (0.084–0.998)	*0.041*
Metastasis	3.579 (1.299–9.862)	0.026	5.795 (1.245–20.964)	*0.025*
Ki-67	4.630 (2.106–10.178)	0.022	0.250 (0.064–0.981)	0.138
AR	0.139 (0.040–0.487)	0.002	0.374 (0.058–2.414)	0.302
Recurrence	16.473 (3.788–71.646)	0.002	0.033 (0.003-–0.326)	*0.003*
B7-H4 (expression)	0.122 (0.022–1.012)	0.045	0.177 (0.012–2.001)	0.213
B7-H4 (intensity)	3.837 (1.773–8.307)	0.007	0.313 (0.114–0.857)	*0.024*

Numbers in italic indicate statistical significance

### B7-H4 abnormally overexpressed on the samples of recurrence TNBC patients after neoadjuvant chemotherapy treatment

**Fig. 3:**
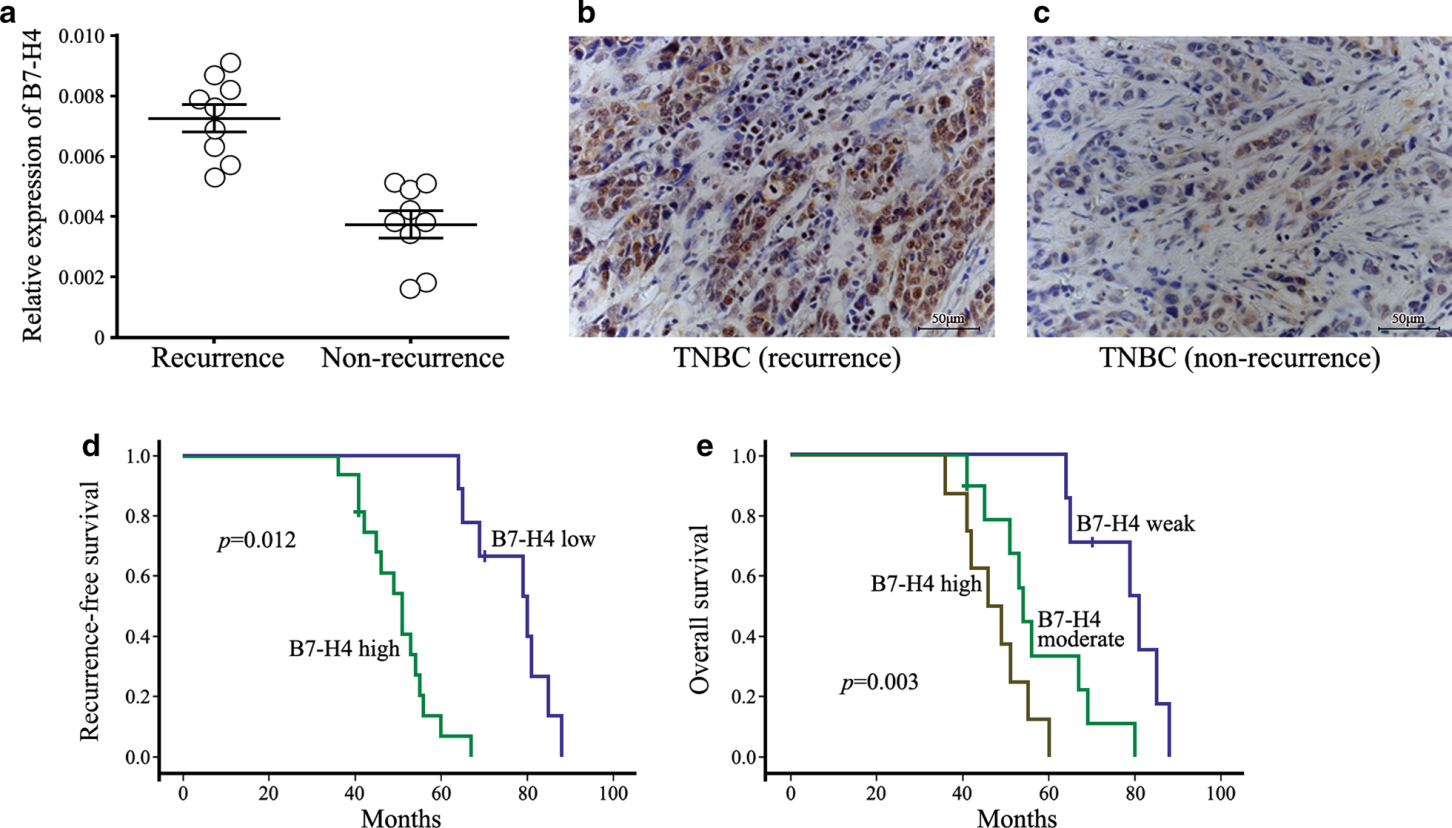
B7-H4 is aberrantly overexpressed in TNBC recurrence patients after neoadjuvant chemotherapy treatment. **a** The expression level of B7-H4 mRNA in the samples of recurrence TNBC patients compared with non-recurrence TNBC tissue samples. **b** B7-H4 immunostaining in the samples of recurrence TNBC patients compared with non-recurrence TNBC tissue samples (**c**). **d** Kaplan–Meier plots of overall survival in recurrence TNBC patients stratified by B7-H4 expression levels. **e** Kaplan–Meier plots of overall survival in recurrence TNBC patients stratified by B7-H4 expression intensity

Interestingly, we found out that there was a positive correlation between the expression status of B7-H4 and patients’ recurrence except all the previous collected results. Many included studies showed that neoadjuvant treatment might differentially affect the patterns of recurrence and overall survival in TNBC patients [20]. Therefore, we analyzed the potential B7-H4 function in patients stratified by the likelihood of their cancer coming back. Results confirmed that B7-H4 mRNA and protein levels were abnormally overexpressed on the samples of recurrence TNBC patients after neoadjuvant chemotherapy treatment (Fig. 3a–c). In this data set, Kaplan–Meier analysis of the local recurrence-free survival between the different groups (higher versus lower expression) showed that women who had higher B7-H4 expression had a significantly increased risk of local recurrence, even after neoadjuvant chemotherapy (Fig. 3d). Especially, when the cohort was divided into Dritter, the increasing rates of local recurrence were noted as the intensities of B7-H4 expression rose (Fig. 3e). In univariate analysis, recurrence-free survival was significantly worse in tumors from patients diagnosed with TNBC which displayed the top strong staining intensity B7-H4 expression (HR = 0.256, 95% CI 0.103–0.635, *p *= 0.003). This is of great clinical significance as disease recurrence in patients diagnosed with cancers of TNBC.

### Anti-proliferative effect of B7-H4 mAb on the chemo-resistant TNBC cell lines

**Fig. 4:**
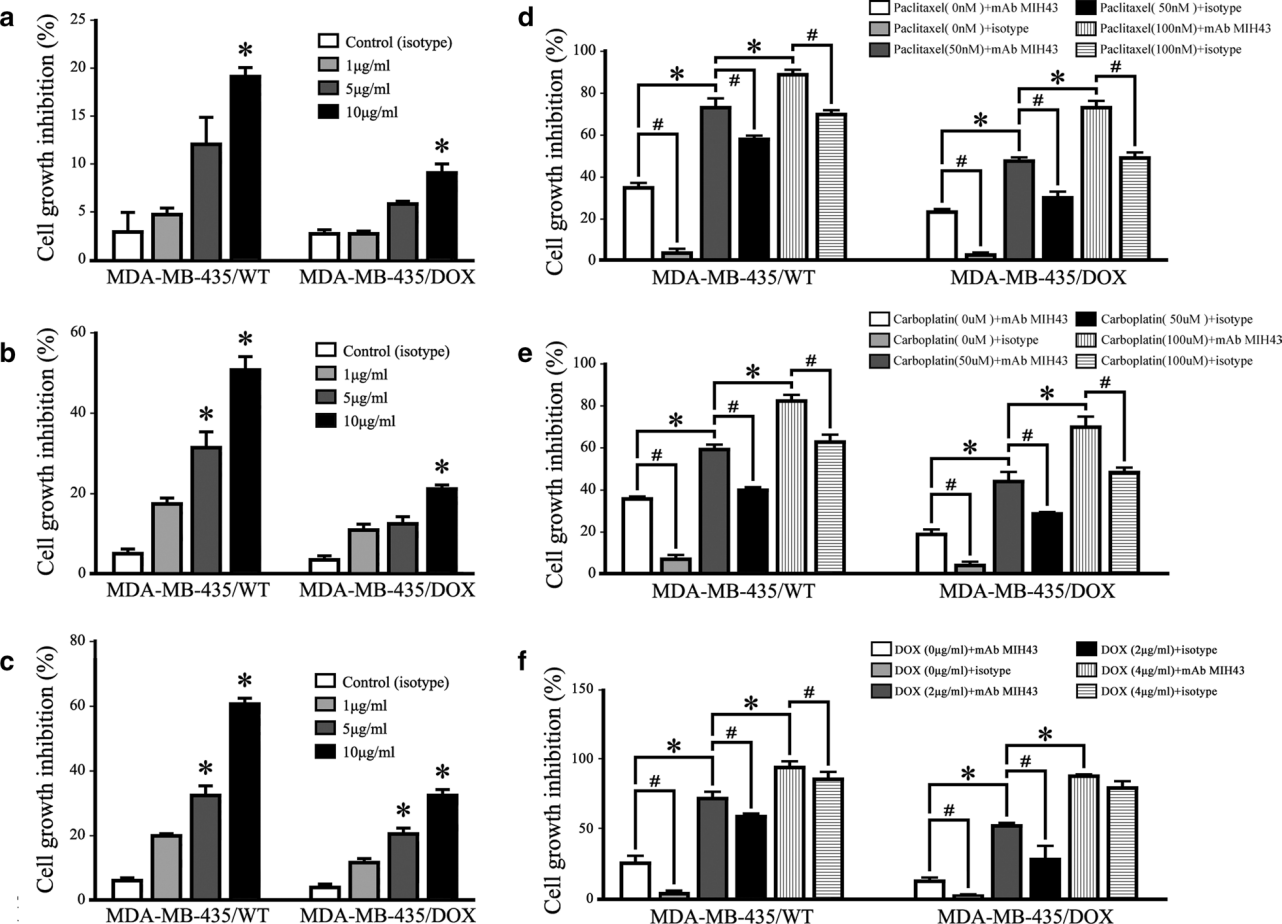
Targeting B7-H4 counteracted the DOX-induced chemo-resistance and increased the sensitivity to DOX, paclitaxel or carboplatin in TNBC cells. To examine the effect of B7-H4 activation on cell viability of MDA-MB-435/WT and MDA-MB-435/DOX cells, different concentrations of anti-B7-H4 antibody were treated for 24 (**a**), 48 (**b**) and 72 h (**c**). Cell proliferation was determined by CCK-8 assay. The combination treatment of mAb MIH43 and paclitaxel (**d**), carboplatin (**e**) or DOX (**f**) increased the cell growth inhibition rate in both MDA-MB-435/WT and MDA-MB-435/DOX cells. Columns, mean of 3 independent experiments done in triplicates, *p < 0.05

Previous results suggested the potential functions of B7-H4 involved in the tumorigenesis and progression of TNBC, even in the recurrence stage, which led us suspect its potential drug resistance capability in the chemo-treatment. Therefore, DOX-resistant MDA-MB-435 cells (MDA-MB-435/DOX) and the parental MDAMB-435/WT cells were plated in 96-well plates at 1 × 10^4^ cells/well and treated with B7-H4 mAb MIH43 at various doses (range 1–10 μg/mL) and compared to treatment with isotype control mAb IgG1. Within the studied concentration range, both 1 μg/mL and 5 μg/mL mAb MIH43 did not show any cytotoxic effects on MDA-MB-435/DOX cells following 24 h incubation, whereas 5 μg/mL mAb MIH43 was shown to be cytotoxic for MDAMB-435/WT cells. Within the tested incubation time prolonged, mAb MIH43 was shown to be more cytotoxic to MDAMB-435/WT cells than to MDA-MB-435/DOX cells upon 48 and 72 h incubation. Monotherapy with mAb MIH43 (10 μg/mL) resulted in over 40% growth inhibition of MDAMB-435/WT cells, and nearly 25% inhibition of MDA-MB-435/DOX cell growth at 48 h (Fig. 4a–c).

In order to study the possible role of B7-H4 in affecting the sensitivity of TNBC cells to DOX, paclitaxel or carboplatin, MDAMB-435/WT and MDA-MB-435/DOX cells were treated with these chemo-drugs together with mAb MIH43 (10 μg/mL) for 48 h. Results showed that treatment of mAb MIH43 with could enhance the sensitivity of both two cell lines to paclitaxel in a significant manner. Similar results were observed for the carboplatin and DOX treatment (Fig. 4d–f).

### B7-H4 silencing increases doxorubicin sensitivity by PTEN/PI3K/AKT pathway

**Fig. 5:**
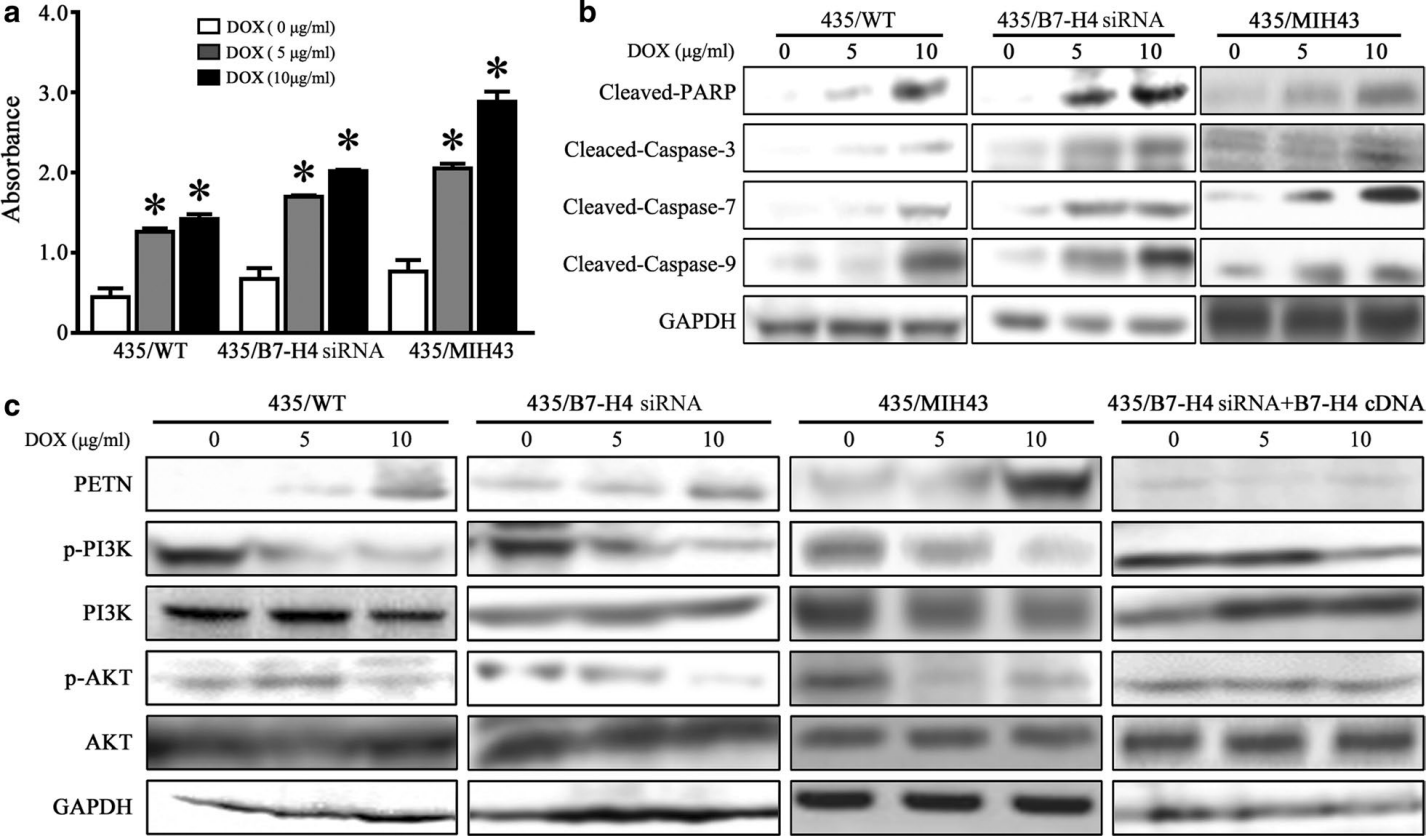
**a** B7-H4 silencing or treatment of mAb MIH43 sensitized breast cancer cells to DOX-induced apoptosis. The percentage of apoptotic cells was investigated by measuring DNA fragmentation by apoptosis-specific ELISA detection kit. **b** B7-H4 silencing or treatment of mAb MIH43 increased apoptosis via promoting cleaved-PARP, cleaved-Caspase-3, cleaved-Caspase-7 and cleaved-Caspase-9 fragmentations. **c** Knockdown of B7-H4 or treatment of mAb MIH43 enhanced the level of PTEN and abolished the phosphorylation level of PI3K and AKT, whereas overexpression of B7-H4 could counteract these effects

Dox is well known for exerting their cytotoxic effects through induction of apoptosis [21, 22], and hence, we investigated whether the increased DOX cytotoxicity observed in B7-H4 knockdown cells could be related to effects on apoptosis. The extent of apoptosis was investigated by measuring DNA fragmentation by apoptosis-specific ELISA detection kit. As shown in Fig. 5a, apoptosis-specific ELISA detection revealed that a dose-dependent of apoptosis was observed in MDA-MB-435/WT cells and the B7-H4 knockdown cells were about 1.5-fold more sensitive to DOX than parental and control cells. Statistical analysis showed that the differences between B7-H4 knockdown and control cells were significant. These results indicate that B7-H4 plays a role in tumor cell apoptosis induced by DOX. To elucidate further the effect of B7-H4 on DOX resistance, MDA-MB-435/WT cells and B7-H4 knockdown cells were treated with 5 μg/mL DOX for 72 h, we observed dramatically increased amounts of cleaved-PARP, cleaved-Caspase-3, cleaved-Caspase-7 and cleaved-Caspase-9 fragmentations in B7-H4 siRNA-transfected cells than in control MDA-MB-435/WT cells (Fig. 5b).

Previous studies have demonstrated that B7-H4 activation leads to abnormally the down-regulation of the AKT pathway in EBV-positive B-cell lymphoma cells [23]. The physiological function of phosphatase and tensin homologue (PTEN), a lipid phosphatase, is a frequently mutated tumor suppressor gene that opposes the PI3K/AKT pathway through dephosphorylation of phosphoinositide-3,4,5-triphosphate [24, 25]. Therefore, we investigate whether B7-H4 was involved by targeting PTEN through the PI3K/Akt signaling pathway. As seen in Fig. 5c, the silencing of B7-H4 induced a dramatic increase in the expression of PTEN both in untreated and DOX-treated cells. Furthermore, the phosphorylation level of p-PI3K was dramatically repressed in B7-H4 silenced cells with reduced Akt phosphorylation (Fig. 5c).

To validate this conclusion, we evaluated whether overexpression of B7-H4 was sufficient to activate PI3K/Akt pathway. B7-H4 cDNA was transiently transfected into B7-H4 knockdown cells 435-siB7-H4 (Fig. 5c, right) and then the phosphorylation level of both PI3K and Akt increased with the B7-H4 expression, further confirming an important role of B7-H4 in regulating the PI3K/Akt signaling pathway.

## Discussion

Evading the antitumor immunity is crucial for the development and progression of cancer [26, 27]. Understanding the dynamic interaction between tumors and the immune system is impendent for the advance of a new cancer immunotherapy [28, 29]. B7-H4 was identified to be one of the fellow B7 family members in 2003, and shown to bind a currently unknown receptor(s) on activated T cells thus resulting in inhibition of T cell effector function in vitro [13, 30]. B7-H4 mRNA is widely expressed in nonlymphoid tissues [31], but its protein expression is largely absent in most normal human somatic tissues, with the exception of epithelial cells from the female genital tract, lung, kidney and pancreas [30]. Abnormal overexpression of B7-H4 was reported in a variety of malignancies including lung, ovarian, breast, prostate, and esophageal cancers [32–36]. B7-H4 is a ligand within the B7 family that has been implicated as a negative regulator of T-cell-mediated immunity, and its expression was inversely related the number of tumor infiltrating T cells [37, 38]. Although the mechanism is still not clear, it is proposed that B7-H4 might employ to evade the host immune system by regulating the differentiation of T cells. The most current literature supports that B7-H4 is a potential negative prognostic indicator for many tumors and could aid in transforming pre-cancerous cells and then protecting them from immunosurveillance [39]. However, in Rahba’s study, he found that reduced MHC I expression and granzyme B expression in CD8^+^T cells infiltrating tumors were detected in B7-H4^−/−^ background mice, together with other evidence showed that B7-H4 expression was not necessary for tumor development but could limit the mammary tumor growth [34, 40]. Collectively, the role of B7-H4 in immune evasion in the cancer microenvironment is yet to be elucidated, especially in breast cancer. Therefore, we want to further study the expression pattern of B7-H4 in TNBC and its potential mechanism.

In this study, we used Cancer Genome Atlas Microarray Database to predict that B7-H4 transcript was dominantly increased in the subtype of invasive breast carcinoma. In the following study, we found that B7-H4 expression was elevated in TNBC cell lines and TNBC patients compared with non-TNBC cells and adjacent non-tumor tissues breast cancer patients and that increased B7-H4 expression was associated with advanced TNM stage and the tendency of metastasis and recurrence. In addition, there was a reverse correlation between the expression of B7-H4 and AR and the outcome of patients. These findings indicate that B7-H4 expression might be linked to more aggressive subtypes of breast cancer and are consistent with previous studies reporting that B7-H4 expression is associated with a poor prognosis in oral squamous cell carcinoma [41], non-small cell lung cancer [42], renal cell carcinoma [43], and glioma [44]. TNBC is a very aggressive subtype of breast cancer due to its lack of the hormonal receptors as well as HER-2 and thus unresponsive to hormonal therapies such as ER/PR antagonists or trastuzumab therapies. Till now, chemotherapy is still the first line treatment of TNBC, however, resistance, relapse, poor response rate and toxicity are common companies associated with chemotherapeutic drugs. Although Leong et al. [45] devised an antibody–drug conjugates against B7-H4 to treat patient-derived xenograft models of triple-negative breast cancer, however, whether chemotherapy could upregulate B7-H4 upregulation and lead to cancer cells become drug-resistant have not been completely studied and reported yet. In our study, we found that B7-H4 expression levels were abnormally overexpressed on the samples of recurrence TNBC patients after neoadjuvant chemotherapy treatment, which indicated that neoadjuvant chemotherapy might induce B7-H4 upregulation. And the increasing rates of local recurrence were noted as the intensities of B7-H4 expression strengthened. Therefore, it might be highly suggested that blocking B7-H4 could be an alternative to to increase the efficacy and reduce the toxicity of the chemotherapeutic drugs.

B7-H4 is a well-defined transmembrane protein, containing one signal peptide and hydrophobic transmembrane domain uniquely anchored to the cell membrane via a GPI linkage [13, 46, 47]. However, some tumor cells were shown to express B7-H4 protein in different subcellular distributions [48, 49]. In our study, we found that B7-H4 protein was shown intense circumferential membranous and cytoplasmic expression in most TNBC cells. So far, the mechanisms and functional implications of B7-H4 subcellular localization remain unclear. Therefore, we selected typically monoclonal antibody MIH43 of B7-H4 for detecting its potential functions. Among the selected TNBC cell lines, the human MDA-MB-435 cell line used in our study was testified to have the highest B7-H4 expression and closely similar to those with MDA-MB-231 and MDAMB-468 breast cancer cells [50, 51]. In this study, we examined the role of B7-H4 in doxorubicin resistance of TNBC cells. Results showed that mAb MIH43 resulted in increased cell growth inhibition of DOX-resistant MDA-MB-435 cells and the parental MDAMB-435/WT cells. In addition, treatment of mAb MIH43 with could enhance the sensitivity of both two cell lines to paclitaxel in a significant manner. Our findings show that targeting B7-H4 could counteract cellular resistance to doxorubicin, and increase the sensitivity to paclitaxel in TNBC cells. Furthermore, in attempts to elucidate the mechanisms underlying the observed effects, we obtained the evidence from a series of functional experiments.

In order to completely elucidate the function of B7-H4, the knockdown and overexpression of the B7-H4 plasmids were constructed respectively. Our finding showed that the apoptotic rate was increased 1.5-fold of TNBC cells sensitive to doxorubicin when B7-H4 was knockdown. Previous investigations showed that breast cancer with axillary lymph node metastases was associated with the abnormal suppressor gene PTEN, which could play a negative regulatory role in the PI3K/Akt signaling pathway [52, 53]. Furthermore, Basho et al. [54] reported that the PI3K/Akt pathway is the major frequently dysregulated pathways in TNBC, which favors the metaplastic TNBC for its transcriptional profiling as the mesenchymal subtype. Interestingly, we found that downregulation of B7-H4 restored the expression of PTEN and reduced phosphorylation of both PI3K and AKT, whereas overexpression of B7-H4 activated PI3K/AKT signaling. This may explain why the B7-H4 knockdown TNBC cells became more prone to doxorubicin-induced apoptosis, and the findings are in accordance with reports showing induction of apoptosis following a blockade of PTEN signaling in multiple cancers [55–57]. On the basis of these findings, we investigated the effects of B7-H4 knockdown on PTEN/PI3K/Akt-regulated genes involved in mitochondrial-pathway apoptosis. The increased expression of cleaved-PARP, cleaved-Caspase-3, cleaved-Caspase-7 and cleaved-Caspase-9 fragmentations in B7-H4 siRNA-transfected cells than in control MDA-MB-435/WT cells, whereas the effects could be counteracted by overexpression of B7-H4.

## Conclusion

Our study investigating the role of B7-H4 in TNBC development and progression showed that B7-H4 was highly expressed in TNBC patients and cells and was associated with a poor prognosis and metastasis. Furthermore, our data indicated that neutralizing antibody against B7-H4 significantly decreased tumor cell viability in vitro, and the protein confers resistance to doxorubicin by reducing the sensitivity of breast cancer cells to apoptosis, mediated via the PTEN/PI3K/Akt pathway. Collectively, these findings provide new insight into the role of B7-H4 in TNBC, which might serve as a prognostic biomarker indicative of poor outcomes and be an effective therapeutic target of TNBC treatment.

## Additional files

**Additional file 1: Figure S1.** (A, B) The effects of B7-H4 knockdown and overexpression was confirmed by real-time PCR and western blotting, respectively. (C) The growth inhibition was influenced after B7-H4 downregulation in MDA-MB-435 WT and MDA-MB-435/DOX cells, respectively.

**Additional file 2: Figure S2.** (A, B) The expression level of B7-H4 protein in various breast cancer cell lines was evaluated using western blotting. The data are presented as the mean ± SD.
