# Supplementary figures and images for: B7-H4 overexpression contributes to poor prognosis and drug-resistance in triple-negative breast cancer
Source: Cancer Cell Int. 2018 Jul 13;18:100. doi: 10.1186/s12935-018-0597-9 (PMC6044050; doi:10.1186/s12935-018-0597-9)

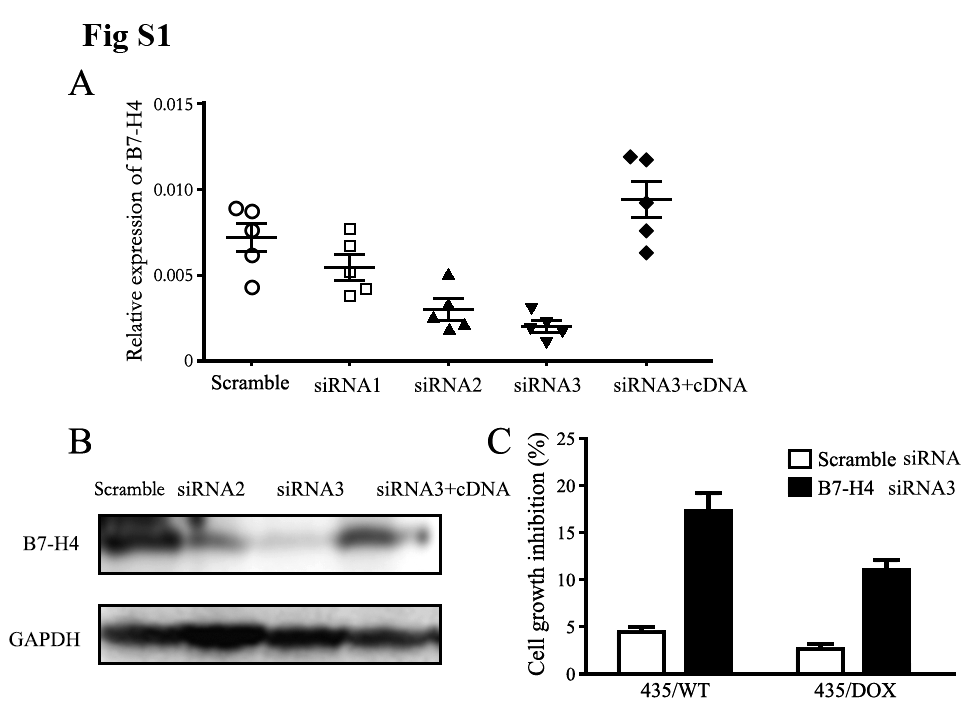

Supplement: Supplementary file 1 — Additional file 1: Figure S1. (A, B) The effects of B7-H4 knockdown and overexpression was confirmed by real-time PCR and western blotting, respectively. (C) The growth inhibition was influenced after B7-H4 downregulation in MDA-MB-435 WT and MDA-MB-435/DOX cells, respectively. [file 12935_2018_597_MOESM1_ESM.tif]

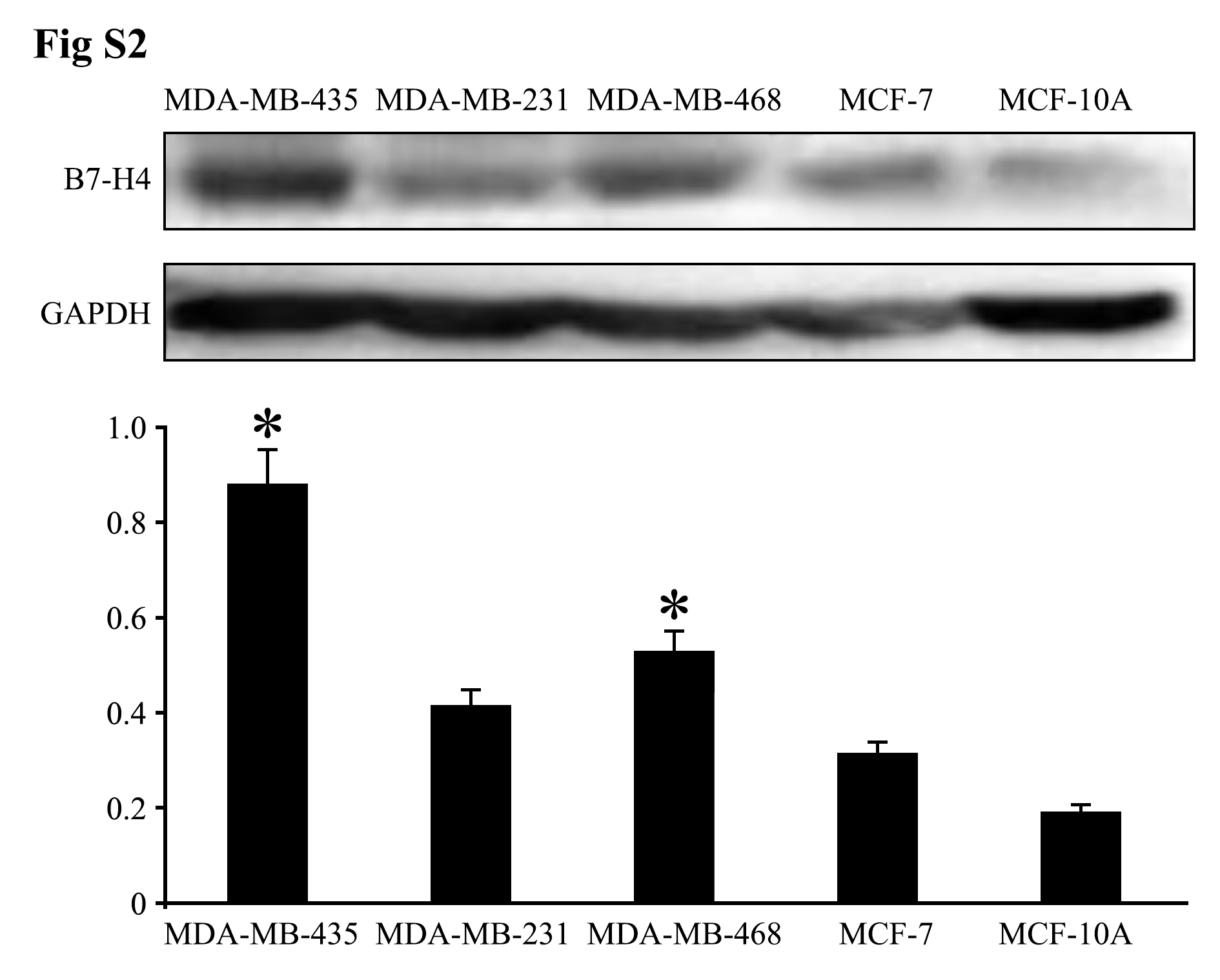

Supplement: Supplementary file 2 — Additional file 2: Figure S2. (A, B) The expression level of B7-H4 protein in various breast cancer cell lines was evaluated using western blotting. The data are presented as the mean ± SD. [file 12935_2018_597_MOESM2_ESM.tif]
